# The non-arthroplasty hip registry of the UK: a decade on

**DOI:** 10.1093/jhps/hnad046

**Published:** 2023-12-21

**Authors:** Christian Smith, Vikas Khanduja, Ajay Malviya

**Affiliations:** Guy’s and Thomas’, London, UK; Cambrige University Hospital NHS Trust, Cambridge, UK; Northumbria Healthcare NHS Foundation Trust and Newcastle University, UK

Last year, with the publication of the 2022 annual report [1], the UK Non-Arthroplasty Hip Registry (NAHR) celebrated its 10-year milestone since it started collecting data regarding patient demographics, peri- and intra-operative details and outcomes following hip-preserving surgery. The NAHR was conceptualized in 2011 and commenced gathering data on hip preserving and non-arthroplasty operations in the UK within a year of its proposal. The primary aim of the registry is to collect longitudinal data on multiple facets of hip preservation surgery with the hope of steering the future of managing hip pathologies which don’t warrant joint replacement surgery and reducing the risk of patients developing hip arthrosis [2], and its associated significant socio-economic burden. Funding for the running costs of the NAHR was initially provided by the British Hip Society, but currently it is independent and receives support from industry and with the help of research grants. This money helps fund future research and development within the registry and subspeciality.

Hip preservation surgery has evolved over the years, with novel and improved techniques, utilization of better implants and embracing enhanced technology; and the NAHR has evolved with it. The current minimum data set (MDS 3.0) collects data on an ever-expanding plethora of intra-operative details including those on anchor usage, labral reconstruction and grafting, and revision procedures. Unlike other non-arthroplasty registries such as the Danish hip arthroscopy registry (DHAR), North American HipSTR registry and various other smaller institution data collection collaboratives, the NAHR is the only one to collect data on all hip non-arthroplasty surgery on a national scale, including soft tissue procedures and pelvic and femoral osteotomies [3].

There are numerous benefits of orthopaedic registries regarding patient outcomes, surgeon regulation and implant scrutiny; including the agility to establish an active surveillance to determine the safety of procedure during a pandemic [4]. Submission of data to the NAHR is performed on a voluntary basis by the majority of surgeons involved, unlike the national joint registry, where data submission is mandatory. As a result, complete surgeon compliance in contributing data to the NAHR has been difficult to achieve at a national level. Currently, the NAHR has 113 contributing surgeons and has recorded over 18 000 individual patient pathways. This makes it the largest registry in the world of its type and continues to grow year on year. The unmonitored use of medical implants is becoming more and more controversial. An investigation into how UK Healthcare oversees and responds to patient harm from drugs and medical devices resulted in the publication of the 2020 Cumberlege report. One of the key recommendations of this report was that all medical implants should be recorded on a central database, where patients can be identified by the type of implant they have received if necessary. Such recommendations strengthen the argument of mandating submission of data to the NAHR.

The NAHR continues to show that non-arthroplasty procedures are highly successful for a wide range of hip pathologies. Both osteotomies of the pelvis and femur, and hip arthroscopy for femoroacetabular impingement have shown significant improvements in collected patient-reported outcome measures (PROMS) at 6 months, and maintenance, if not continued improvement, at 12 months after surgery [3, 5–7]. There are other key findings borne out by the NAHR which have influences hip preservation surgery, an example of this is the effect of the patients age on outcomes after hip arthroscopy and pelvic osteotomies. Analysis of the registry data showed that outcomes following hip preservation surgery become increasingly unpredictable after the age of 40. This reaches a point at around 50 years of age where the benefits of the operations become less apparent, and there is a greater chance of rapid deterioration, sometimes after a brief period of improvement, to a point worse off than before their operation. As a result of the registry’s finding, there is improved patient selection helping to choose those who are likely to benefit from the surgery. Surgeons do not exclude patients over 40 as a result, but have the evidence to be more cautious in offering hip preservation surgery to this cohort, often with greater discussion of the risk–benefit balance and a more guarded prognosis to aid an honest and open-shared decision-making process.

Besides the analysis of patient outcomes, the NAHR has looked to enhancing the future of hip preservation surgery in the UK and on a wider scale. The NAHR has appointed a research fellow to explore use of artificial intelligence and machine learning to help predict long-term outcomes following hip preservation surgery. A feasibility study into embedding research into registries has already demonstrated early success with its pilot study on the effectiveness of outcome teams. Best practice guidelines for managing femoroacetabular impingement and hip dysplasia have also been produced, aiming to educate, streamline care and remove variability from the management of these conditions.

Although the NAHR has achieved a vast amount, surpassing its original goals when it was conceived, there are still numerous improvements which can be made. The DHAR [8] collects a greater variety of peri-operative data and PROMS, and has greater compliance for post-operative PROMS completion. NHS England has unveiled plans to help support national registries and improve surgeon and patient compliance. The NAHR has already performed a pilot study proving that patient compliance can be improve significantly with the use of local administrative support. This strengthens the argument for hospitals investing in ‘Outcome teams’, to assist the registries in enhancing data capture. It is only through improved data collection that the NAHR will continue to have such an influential effect on the future of hip preservation surgery.
